# CARDIORESPIRATORY FITNESS ASSOCIATED TO TEENAGERS’ FAT: VO_2MAX_ CUTOFF POINT

**DOI:** 10.1590/1984-0462/;2019;37;1;00017

**Published:** 2019-01-07

**Authors:** Leandro Smouter, André de Camargo Smolarek, William Cordeiro de Souza, Valderi de Abreu de Lima, Luis Paulo Gomes Mascarenhas

**Affiliations:** a Universidade Estadual do Centro-Oeste, Irati, PR, Brazil.; b Universidade do Contestado, Canoinhas, SC, Brazil.; c Universidade Federal do Paraná, Curitiba, PR, Brazil.

**Keywords:** Pediatric obesity, Association, Cardiorespiratory fitness, ROC curve, Obesidade pediátrica, Associação, Aptidão cardiorrespiratória, Curva ROC

## Abstract

**Objective:**

To associate the Maximal Oxygen Uptake (VO2max) with body fat percentage (%BF), and to establish the best VO2max cutoff point for predicting risk %BF in teenagers.

**Methods:**

This study was carried out with 979 subjects aged 10 to 18.8 years, 556 (56.8%) girls. The 20 m shuttle run protocol determined the VO2max, which was analyzed in quintiles and in a numeric scale. Cutaneous fold equations determined the %BF, later classified as risk to health/obesity when >25 in girls and >20 in boys. Regression method was used - *Odds Ratio* (OR) and Receiver Operating Characteristics Curve (ROC curve) with α <5%.

**Results:**

From the total number of valid cases, 341 (65.6%) girls and 202 (53.2%) boys presented %BF of risk, and a larger proportion of %BF of risk was observed in the 1st quintile of the VO2max for both genders. There was inverse association between VO2max and %BF of risk from the 4th quintile (OR 1.84, 95%CI 1.05-3.24) until the 1st quintile (OR 4.74, 95%CI 2.44-9.19) for girls, and from the 2nd quintile (OR 2.99, 95%CI 1.48-6.00) until the 1st quintile (OR 5.60, 95%CI 2.64-11.87) for boys. As analytic highlights, VO2max Cutoff points for prediction of %BF of risk were ≤40.9 mL/kg-1/min-1 (AUC: 0.65; p<0.001) for girls and ≤44.8 mL/kg-1/min-1 (AUC: 0.66; p<0.001)for boys..

**Conclusions:**

VO2max was inversely associated to the %BF, and VO2max cutoff points for prediction of %BF of risk are important results to generate action to fight early obesity.

## RESUMO

**Objetivo:**

Associar o Consumo Máximo de Oxigênio (VO_2máx_) com o % de Gordura Corporal (%_GC_) e estabelecer o melhor ponto de corte do VO_2máx_ para a previsão do %_GC_ de risco em adolescentes.

**Métodos:**

Estudo realizado com uma amostra de 979 sujeitos entre 10 e 18,8 anos, sendo 556 (56,8%) meninas. O protocolo *shuttle run* de 20 m determinou o VO_2máx_, que foi analisado em quintil e escala numérica. Equações de dobras cutâneas determinaram o %_GC_, posteriormente classificado como risco à saúde/obesidade quando >25, para meninas, e >20, para meninos. Utilizou-se regressão - *Odds Ratio* (OR) e Curva Característica de Operação do Receptor (curva ROC) com α <5%.

**Resultados:**

Do total de casos válidos, 341 (65,6%) meninas e 202 (53,2%) meninos apresentaram %_GC_ de risco, e constatou-se maior proporção do %_GC_ de risco no 1º quintil do VO_2máx_ para ambos os sexos. Houve associação inversa entre VO_2máx_ e %_GC_ de risco do 4º quintil (OR 1,84, IC95% 1,05-3,24) até o 1º quintil (OR 4,74, IC95% 2,44-9,19) para meninas, e do 2º quintil (OR 2,99, IC95% 1,48-6,00) até o 1º quintil (OR 5,60, IC95% 2,64-11,87) para meninos. Pontos de corte do VO_2máx_ para previsão do %_GC_ de risco ≤40,9 mL/kg^-1^/min^-1^ (*Area Under the Curve* (Área Sob a Curva) - AUC: 0,65; p<0,001) para meninas e ≤44,8 mL/kg^-1^/min^-1^ (AUC: 0,66; p<0,001) para meninos foram destaques analíticos.

**Conclusões:**

VO_2máx_ esteve associado inversamente ao %_GC­_, e os pontos de corte do VO_2máx_ para a previsão do %_GC_ de risco são resultados importantes para ações de combate à obesidade precoce.

## INTRODUCTION

At the global level, considering countries and territories, there is a history of increase in access to and quality of personal health care between 1990 and 2015.^1^ However, the prevalence of obesity is a factor of worldwide concern, as it grows faster than the access to health care by the population, reaching alarming estimates by the year 2030.^1^ ^,^ ^2^ At the top of the list, the United States, with 65 million cases, and the United Kingdom with 11 million, are the regions with the highest predictions of obese adults by the year 2030.^2^ In its course, obesity is expected to increase the burden of cardiovascular diseases, with noteworthy figures between 6 and 8.5 million cases of diabetes, and between 5.7 and 7.3 million stroke cases, and the same extends to other regions of the globe.^2^

Specifically in the Americas, concern about the increasing numbers of obese adults projected for the coming years is evident.^3^ Agreements between North American countries and policymaking in Latin America are some of the actions being taken to fight early obesity in teenagers.^4^ ^,^ ^5^ This is because obesity is a modifiable risk factor, which begins mainly in adolescence, a phase in which the imbalance in body fat rates begins to occur.^6^ Most of the individuals who will be adults in 2030 are adolescents today, so keeping body fat controlled below health risk levels is essential at this stage.

A 26-year prospective follow-up study of 770 subjects, ages 5 to 20, revealed that the elevated triglyceride rate in adolescence has led to cardiovascular events and increased rates of type II diabetes mellitus in adulthood, and triglyceride is an important component of the body fat rate.^7^ It also evidences that adolescents with Body Fat Percentage (%_BF_) above health risk levels are 2.2 times more likely to develop cardiovascular complications.^8^

Adolescence is characterized by morphological, physiological, and behavioral changes influenced by exogenous factors, such as physical, social, and environmental.^9^ Cardiorespiratory Fitness (CRF) increases naturally as time progresses in adolescence, and extends over a longer period in boys.^10^ However, the typical transformations of adolescence are irregular, and contribute to the fact that the natural increase of CRF is not adequately sustained in some individuals, who develop and maintain insufficient CRF and excessive %_BF_ which, if combined, can lead to obesity.^10^

CRF based on Maximum Oxygen Consumption (VO_2max_) and %_BF_ are important health markers.^11^ Although the odds of presenting %_BF_ that is a health risk are 3.2 times higher in adolescents with low VO_2max_,^12^ precise VO_2max_ cutoff points for predicting risk %_BF_ are still not well defined in this age stratum. Thus, this study aimed to associate VO_2max_ with %_BF_ and to establish the best VO_2max_ cutoff point for predicting %_BF_ considered as a health/obesity risk in adolescents.

## METHOD

This is a school-based cross-sectional study developed between 2010 and 2012. Participants were 979 subjects aged between 10 and 18.8 years old from the city of Curitiba, Paraná, Brazil; 556 (56.8%) girls and 423 (43.2%) boys. The selection was made in three stages:

- Analysis by conglomerate, in which five regions of the municipality (north, south, east, west, and central) were selected.
- Simple random probabilistic analysis, in which 12 schools were selected, considering the pre-selected regions.
- Selection of participants corresponding to each eligible institution on a random basis.

In addition, four exclusion criteria were considered: physical impairment in the evaluation of CRF; fat loss accelerator drugs; lack of consent to participate; and chronological age outside the 10-19 years age range.

In addition to the chronological age for the calculation of CRF, sex and biological maturation variables were evaluated and controlled, considering their influence on VO_2max_ and on %_BF_ during adolescence.^13^ Age was identified based on date of birth in the participants’ school records. The records also provided information on the first controlled factor: sex (female or male). For the control of sex in the analyzes, sex-separated results were performed and presented.

The Tanner criteria^14^ were considered for the second controlled factor: biological maturation, characterized as BM_TANNER_. In order to evaluate it, the participant’s self-assessment was adopted, in which images pre-established by Tanner^14^ were presented to the subjects; the participants conducted a self-analysis and indicated the stage (I, II, III, IV or V) in which they were at the moment. Then, the stages were regrouped, according to Tanner,^14^ in pre-pubertal (stage I), pubertal (stages II, III and IV), and post-pubertal (stage V), thus forming a three-leveled factor considered in calculations and analysis.

The anthropometric variables evaluated were: Stature (STA), Body Mass (BM), Tricipital Skin Fold (TRSF) and Subscapular Skin Fold (SSSF). To quantify the STA in meters (m), the participant was positioned in accordance with the Frankfurt plan, without footwear, in a WCS^®^ portable stadiometer (Curitiba, Paraná, Brazil) with a precision of 0.1 cm, with the arithmetic mean of two invariant measurements being 0.2 cm of the final STA value.^15^ BM was quantified in kilograms (kg); for this, the participant, without footwear and in light clothing, was positioned on the platform of a PLENA^®^ digital scale (Bom Retiro, São Paulo, Brazil) with an accuracy of 100 g, with the arithmetic mean of two invariant measurements being 0.2 cm of the final BM value.^15^ For the quantification of TRSF and SSSF in millimeters (mm), the anatomical repair points of the protocol by Slaughter et al.^16^ were used for the positioning of a CESCORF^®^ plicometer (Porto Alegre, Rio Grande do Sul, Brazil) with an accuracy of 0.1 mm, with the arithmetic mean of three invariant measurements being 0.2 mm for each fold of the final values of the TRSF and SSSF.

CRF was evaluated by means of the VO_2max_ with the 20 m shuttle run field protocol of Léger and Lambert.^17^ This protocol evaluates VO_2max_ indirectly through the subject’s fitness stage. More details of the test can be obtained in another study.^18^ In short, the subjects moved back and forth on a 20 m course on the sports court, each displacement being commanded by a sound signal that started at 8.5 km/h and accelerated 0.5 km/h in each stage. As the subject could no longer keep pace, the last complete displacement was considered to determine the reference stage in the calculation of the VO_2max_ with the following Equation 1:

$${VO}_{2max.}\;\;=\;31.025\;+\;(3.238\;x\;A)\;-\;(3.248\;x\;B)\;+\;0.1536\;(B\;x\;A)$$ (1)

Where:

A: speed in the last stage;
B: age, in years.

The Léger and Lambert protocol^17^ is a reliable estimator of VO_2max_ in adolescents; t was compared with the gold standard gas analyzer method in a validity research^19^ (r=0.93) and in a revaluation study^20^ by meta-analysis (*r*_p_=0.78). The estimated value of VO_2max_ can be presented in liters per minute (L/min) or relative to the subject’s BM, in milliliters per kilogram per minute (mL/kg^-1^/min^-1^). For the sake of accuracy, the mL/kg^-1^/min^-1^ unit was used.^17^ In addition, VO_2max_ was analyzed in its division by five balanced groups (quintile) and in numerical scale, respecting the criteria of the analytical methods used.

For the calculation of %_BF_, the values of TRSF and SSSF were used, as well as values for each stage of BM_TANNER_, according to Slaughter et al.,^16^ in the following Equation 2:

$${\%}_{BF}=\left[\begin{array}{c} Pre-pubertal[1.21\left(TRSF+SSSF\right)-0.008\;{\left(TRSF+SSSF\right)}^{2}-1.7\\ Pubertal[1.21\left(TRSF+SSSF\right)-0.008\;{\left(TRSF+SSSF\right)}^{2}-3.4\\ Post-pubertal[1.21\left(TRSF+SSSF\right)-0.008\;{\left(TRSF+SSSF\right)}^{2}-5.5 \end{array}\right.$$ (2)

Where:

TRSF: Tricipital Skin Fold;
SSSF: Subscapular Skin Fold.

In adolescents, the equations by Slaughter et al.^16^ presented a good correlation (r=0.90) with the gold standard method for the evaluation %_BF_ - *Dual Energy X-Ray Absorptiometry*,^21^ not causing financial inflation to the research and being recommended for use with this age group.^22^

Age-specific cutoff values for age and gender studied by Lohman,^23^ and recently also considered by Pelegrini et al.,^6^ were used in the classification of the %_BF_, considering health risk/obesity values >25 for girls and >20 for boys.

Microsoft Office Excel^®^ 2010 (Redmond, Washington, United States) and MedCalc^®^, versão 11.3 (Oostende, West Flanders, Belgium), were the software used to tabulate the data with double typing for analytical procedures. Descriptive indicators of mean, standard deviation, median, minimum value, maximum value, absolute frequency, relative frequency, and quintile were used. Two analytical methods were applied: binary logistic regression - *Odds Ratio* (OR), with BM_TANNER_ correction in block; and Receiver Operating Characteristic Curve (ROC curve). All analyzes considered α <5%.

Free and informed consents were used for consent by the participants’ and volunteers’ legal guardians. All the ethical aspects provided in Resolution 196/1996, in force during the study period, current Resolution No. 466/2012 of the Brazilian National Health Council, were observed. The study was approved by the Research Ethics Committee of Universidade do Paraná, approved under Protocol No. 0137.0.208.000-07.

## RESULTS

The results presented below correspond to the 900 valid cases, 520 (57.7%) females and 380 (43.3%) males. Thus, 79 individuals (8.0%) were considered losses, as there were missing data essential to the analyzes. The mean age was 13.7±2.1 and 13.9±2.0 years for girls and boys, respectively. The descriptive results, according to the variables studied and the sex, are presented in Table 1.

**Table 1:** Descriptive data according to the sex of the adolescents.

Variable	Sex							
	Females (n=520)				Males (n=380)			
	Ẋ ±SD	Md	Min.	Max.	Ẋ ± SD	Md	Min.	Max.
Age (years)	13.7±2.1	13.6	10	18.8	13.9±2.0	13.9	10.3	18.4
BM_TANNER_*	3.4±0.9	4	1	5	3.4±1.0	4	1	5
STA (m)	1.56±0.08	1.56	1.27	1.82	1.62±0.14	1.63	1.31	1.96
BM (kg)	50.5±11.7	49.8	24.9	96.5	54.4±15.1	54.3	25.7	123.9
TRSF (mm)	15.0±5.3	14.0	5.6	34.0	11.0±5.4	9.6	4.3	31.3
SSSF (mm)	12.3±6.5	10.5	4.5	45.3	9.9±6.6	7.8	4.1	46.5
BF (%)	30.0±9.1	27.9	14.7	65.5	23.3±9.3	20.5	11.3	71.3
VO_2max_**	39.5±5.2	39.3	20.2	65.0	46.8±7.1	46.8	26.8	65.6
VO_2max_ (quintiles)								
1^st^ quintile**	32.4±2.9	33.3	20.2	35.8	36.8±3.0	37.4	26.8	40.6
2^nd^ quintile**	36.8±0.7	36.9	35.9	38.2	43.0±1.2	43.3	40.7	44.9
3^rd^ quintile**	39.3±0.7	39.3	38.3	40.6	46.8±1.1	46.8	45.0	48.9
4^th^ quintile**	42.0±0.8	42.0	40.7	43.8	50.7±1.0	50.9	49.0	52.9
5^th^ quintile**	46.8±3.4	45.7	43.9	65.0	56.7±3.0	56.4	53.0	65.6

Ẋ: arithmetic mean; SD: standard deviation; Md: median; Min.: minimum value; Max.: maximum value; BM_TANNER_: biological maturation; STA: Stature; BM: Body Mass; TRSF: Tricipital Skin Fold; SSSF: Subscapular Skin Fold; BF: Body Fat; VO_2max_: Maximum Oxygen Consumption; *Tanner stage; **mL/kg^-1^/min^-1^.

**Figure 1:**
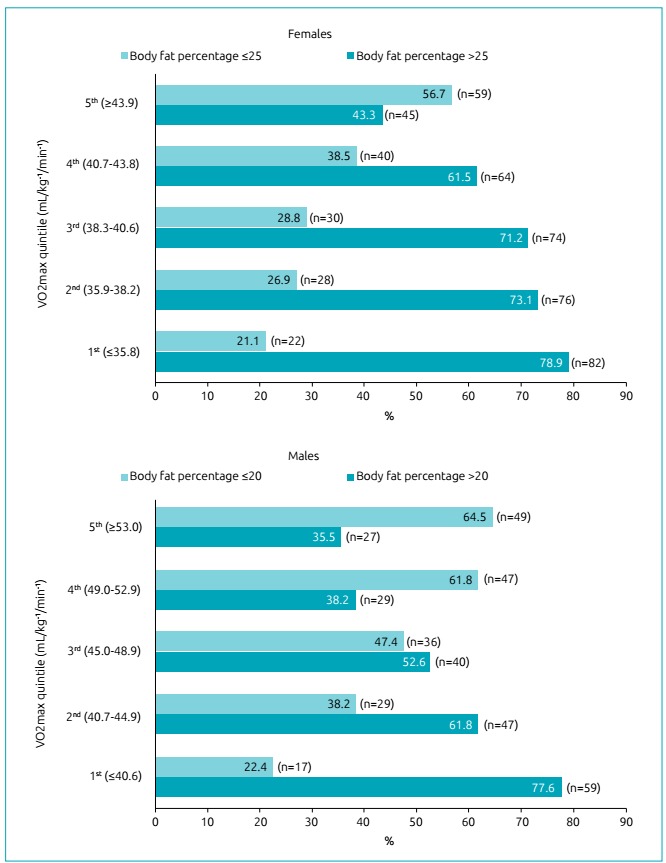
Descriptive analysis of Body Fat Percentage lower and higher than the level of health risk, according to the quintile of Maximum Oxygen Consumption and according to the sex of the adolescents.

For females, 341 (65.6%) individuals had risk %_BF_; for males, this outcome occurred in 202 (53.2%) cases. There was a higher proportion of risk %_BF_ in the 1^st^ quintile of VO_2max_, as well as lower frequency in the 5^th^ quintile for both sexes. In Figure 1, %_BF_ results are presented considering categories lower and higher than the health risk criterion, according to the VO_2max_ quintile.

**Figure 2:**
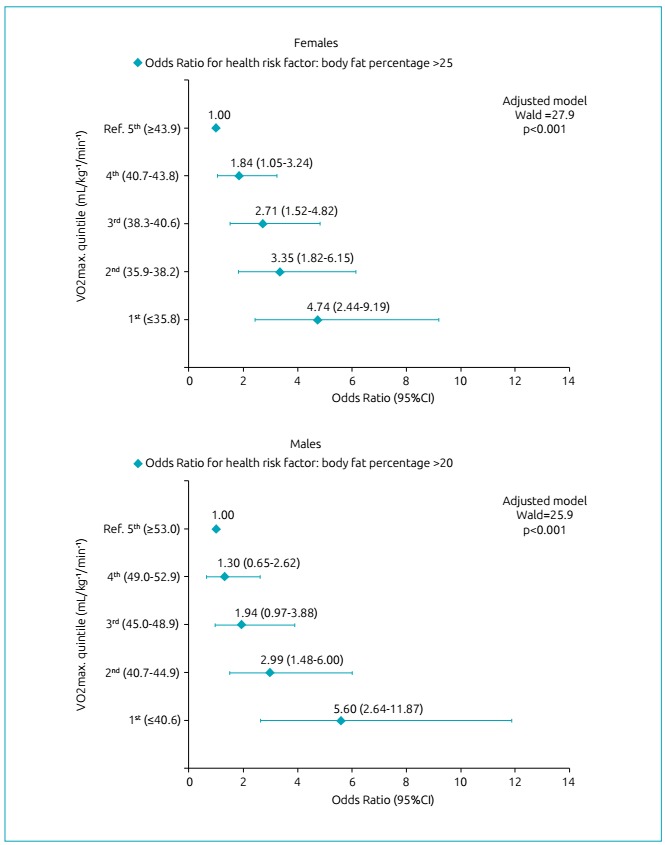
Odds Ratio with 95% confidence interval for the Body Fat Percentage of health risk in adolescents, according to the quintile of Maximum Oxygen Consumption and after adjustment for stage of biological maturation in the binary logistic regression analysis.

The regression models obtained significant adjustments, both for females (Wald: 27.9; p<0.001) and for males (Wald: 25.9; p<0.001). For girls, the OR for risk %_BF_ increased significantly as VO_2max_ decreased, with this trend occurring from the 4^th^ quintile (OR 1.84, 95%CI 1.05-3.24) to the 1^st^ quintile (OR 4.74, 95%CI 2.44-9.19). For boys, this outcome was repeated, but only from the 2^nd^ quintile (OR 2.99, 95%CI 1.48-6.00) to the 1^st^ quintile (OR 5.60, 95%CI 2.64-11.87). In Figure 2, the regression results are presented.

**Figure 3:**
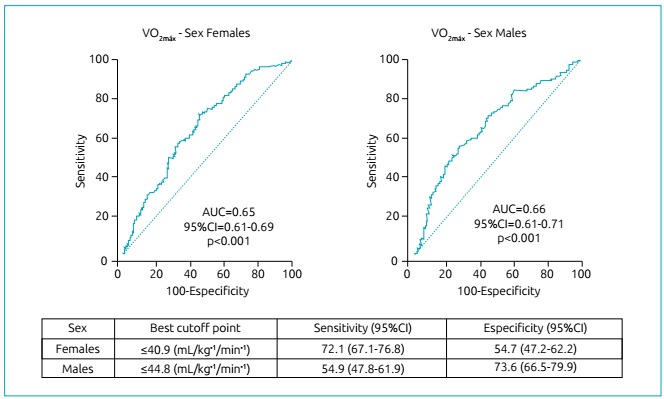
ROC curve analysis, according to sex, and best cutoff point of Maximum Oxygen Consumption for the prediction of Body Fat Percentage above the level of health risk in adolescents.

The VO_2max_ cutoff points agreed with the regression analyzes, that is, the cutoff point ≤40.9 mL/kg^-1^/min^-1^ obtained on the ROC curve for the female sex is within the quintile in which the OR became significant for these subjects. This outcome also occurred for males, since the cutoff point was ≤44.8 mL/kg^-1^/min^-1^. In Figure 3, results of the ROC curve analysis with VO_2max_ cutoff values are presented for the prediction of risk %_BF_.

## DISCUSSION

The present study contributes to the evidence of inverse association (+VO_2max_ = **-**chance of risk %_BF_) between VO_2max_ and %_BF_ in adolescents. It is noteworthy that, although the association occurred in both sexes, in girls, it occurred two quintiles earlier than in boys, implying, from the clinical-functional point of view, different VO_2max_ cutoff values between the sexes. Thus, the best VO_2max_ cutoff point for predicting risk %_BF_ on the ROC curve was lower for girls.

To explain the inverse association between VO_2max_ and %_BF_ in different VO_2max_ quintiles for girls and boys, it can be suggested that there is a higher propensity to iron deficiency in girls than in boys in adolescence,^24^ and since this type of deficit negatively affects the transport of oxygen through the body, CRF would be reduced in girls.^25^ Secondly, it is evident that the %_BF_ is higher in girls than in boys in this age group due to metabolic differences caused by the biological maturation process.^26^ Thus, these two facts also corroborate the lower VO_2max_ result for girls, as well as the lower %_BF_ for boys (Table 1), indicating agreement with the literature.^24^ ^,^ ^25^ ^,^ ^26^

VO_2max_ was significantly associated with risk %_BF_ first in girls, that is, when subjects enter adolescence, VO_2max_ tends to decrease and %_BF_ tends to increase before in girls than in boys (Figure 1). Thus, the OR only points out the most specific quintile in which these associations occur (Figure 2), especially in boys, since the inverse association between VO_2max_ and risk %_BF_ for girls has already been evidenced in a specific research for this gender.^27^ Thus, the data presented in this study agree with such research, in addition to adding that this association also exists for boys.

In the regression analyzes, VO_2max_ values increase the OR for risk %_BF_ to close values in both sexes, but in thresholds for different quintiles (Figure 2), that is, when they reach <43.8 mL/kg^-1^/min^-1^ (4^th^ quintile) for girls and <44.9 mL/kg^-1^/min^-1^ (2^nd^ quintile) for boys. This fact reveals that the determinant of the quintile discrepancy, in which the OR becomes significant between the two sexes, is the physiological and morphological characteristics, since the VO_2max_ values are similar; and the association occurs earlier in girls, because the adipose tissue gain propensity is higher in them in relation to the boys, when there is loss in the CRF in that age group.^28^

In another study, mean values found for VO_2max_ were 41.1 and 44.2 mL/kg^-1^/min^-1­^ for boys and girls, respectively.^26^ These two values are close to the two cutoff points established in the ROC curve for %_BF_ values predicted for girls and boys in the present study (Figure 3). Thus, the previously mentioned research^26^ contributes to emphasize the accuracy of the values found in the two ROC curves in this study, since the authors^26^ also evaluated the %_BF_ with the protocol by Slaughter et al.^16^ and, on that occasion, they found average descriptive values of 25.0% in girls and 20.0% in boys, exactly the cutoff values adopted in the present study for %_BF_ considered as a health risk.

Endogenous sex hormones acting in adolescence can also influence the peripheral constitution of tissues. The hormones estrogen and testosterone are produced by both sexes, but in a disparate way.^13^ On the one hand, there is greater production of estrogen in females; being a facilitator for the deposition of adipose tissue, the concentration of fat in girls is higher.^13^ On the other hand, there is a greater production of testosterone in males; being a determinant for the natural development of muscle mass, the concentration of musculoskeletal mass tends to be higher in boys.^13^ Thus, boys present higher VO_2max_ than girls do in this age group, because VO_2max_ is increased with muscle mass gain. This is a fact that contributes to the cutoff point obtained in the ROC curve being larger for boys (Figure 3).

Insufficient VO_2max_ and risk %_BF_ are associated with low-grade inflammation in the arterial wall in adolescents.^29^ Low-grade inflammation produced by C-reactive protein (CRP) and interleukin-6 (IL-6) cytokine is a predictor of the risk for cardiovascular diseases.^29^ The principle of this statement is that low-grade inflammation caused by CRP and IL-6 mediates obesity, inflammation, insulin resistance and cardiovascular diseases.^29^ ^,^ ^30^

In this sense, %_BF_ below the health risk value helps to control low-grade inflammation in the arterial wall, that is, it contributes to avoid the inflammatory action of CRP and IL-6, whose non action consequently helps avoiding cardiovascular diseases. However, VO_2max_ takes a dual function in the process. On the one hand, its elevation helps to inhibit inflammation by CRP and IL-6 in the arterial wall.^29^ On the other hand, if found in values below the cutoff points presented by the present study (Figure 3), it also helps predicting risk %_BF_, and may be considered as an alert for the control of early obesity in adolescents.

Keeping %_BF_ below the health risk level is important to avoid early obesity, and VO_2max_, at appropriate levels, contributes to this. Activities that lead to maintenance and elevation of VO_2max_, such as exercise and regular physical activity, are essential. In addition, practitioners involved with adolescents’ physical activity practices may use theVO_2max_ cutoff points obtained in the present study (Figure 3) to alert young people to the risk of being susceptible to imbalance in body fat rates, since the shuttle run field protocol is feasible to daily practice.

Some limitations of the study should be pointed out: the first is that the variables VO_2max_ and %_BF_ were studied indirectly. The second is that the VO_2max_ was calculated based on the maximum evaluation, unlike studies in which submaximal evaluations are considered. And the third is that the values of cutoff points established for VO_2max_ in the present study (Figure 3) are valid only for adolescents.

The number of cases studied was in good volume to establish cutoff points for VO_2max_, but research to improve them and corroborate them in the prediction of risk %_BF_ with direct measures will be necessary to evaluate possible uncontrollable divergences in indirect methods. Scaling the ratio of the %_BF_ reduction to the VO_2max_ elevation and how long the VO_2max_ needs to be maintained for the fat oxidation process to improve will also be challenges to future research.

As a conclusion VO_2max_ was inversely associated with %_BF­_ in girls and boys. The VO_2max_ cutoff point for predicting risk %_BF_ was lower for girls (≤40.9 mL/kg^-1^/min^-1^) than for boys (≤44.8 mL/kg^-1^/min^-1^). These results are important for the exchange of relevant information among different nations for the development of programs, guidelines, and promising practices for fighting early obesity, since obesity control is considered one of the priorities of the health field.
